# Adult-Onset White Matter Vanishing Disease With Ovarian Failure in a Salvadoran Patient

**DOI:** 10.7759/cureus.39900

**Published:** 2023-06-03

**Authors:** Sara Loanny Flores Lazo, Jaime Leonardo I Salazar-Orellana

**Affiliations:** 1 Department of Neurology, Instituto Salvadoreño del Seguro Social, San Salvador, SLV; 2 Department of Neurology and Psychiatry, Instituto Nacional de Ciencias Médicas y Nutrición Salvador Zubirán, Mexico City, MEX

**Keywords:** ovarioleukodystrophy, leukodystrophies, white matter demyelination, white matter vanishing disease, ovarian failure

## Abstract

White matter vanishing disease is a type of leukodystrophy common in children with hypomyelination linked with ataxia, all of whom have an autosomal recessive inheritance. There are few reports of late-onset cases associated with ovarian failure. In Mesoamerican populations, this disease is mostly reported in children but rarely in adults. We present a case of a 35-year-old Salvadoran female patient with a history of menometrorrhagia, infertility, slowly progressive gait decline, paraparesis, spasticity, cerebellar ataxia, cognitive impairment with predominant executive dysfunction, learning difficulties, and emotional lability. A T2-weighted brain MRI and fluid-attenuated inversion recovery (FLAIR) images showed bilateral symmetrical areas of hyperintensities in the white matter with multiple foci of cystic degeneration within the hyperintense area. Two pathogenic variants were identified in the EIF2B5 gene. This case is of interest to increase awareness of late-onset leukodystrophies, widen the phenotypic spectrum of the disease, and constitute a valuable report for the international community, since it is a less frequently reported disease.

## Introduction

White matter vanishing disease (WMVD) is a type of leukodystrophy that was initially described in children. Its onset may vary widely, from childhood-onset disease to adult-onset slow progressive disease [1]. Since the first descriptions made by Eicke [2] and Deisenhammer [3], as well as clinical reports on children made by Hanefeld [4], there are few reported cases in Mesoamerican populations in children and none in Central American populations. WMVD is also related to ovarian disturbances such as primary or secondary ovarian failure, and a wide variety of symptoms occasionally precede neurological impairment. These presentations are scarcely described in adult-onset presentations. The rank of severity may range from mild to aggressive. Herein, we present a female patient with adult-onset ovarian disturbances and progressive neurological impairment.

## Case presentation

**Figure 1:**
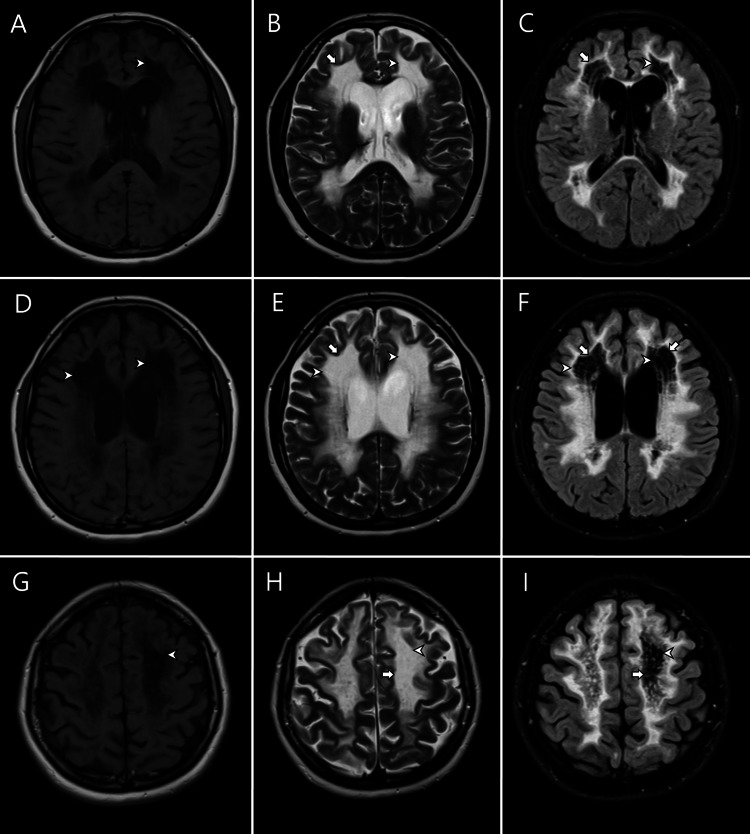
Brain MRI Axial MRI showing bilateral diffuse white matter affection that appears hypointense on T1-weighted magnetic resonance (MR) images (panels A, D, and G) and hyperintense on T2-weighted (panels B, E, and H) and T2-fluid-attenuated inversion recovery (FLAIR) MR images (panels C, F, and I) compared to normal white matter. Also, some lesions isointense to CSF at T1-weighted, T2-weighted, and FLAIR images representing cystic degeneration of white matter can be seen (arrowheads).

We present the case of a 35-year-old female patient, originally from El Salvador, who was referred to our hospital with a two-year history of progressive onset of spastic gait, frequent falls, balance disturbances, learning difficulties, and frequent forgetfulness. Past medical history and neurological and neuropsychiatric background were unremarkable. She did not have any abnormalities during the perinatal, neonatal, or childhood period. Gynecological history reported menarche at the age of 14 years. Since then, she described an abnormal menstrual pattern with irregular cycles and menometrorrhagia and also described multiple failed attempts to conceive a pregnancy. A neurological examination of cognitive functions revealed a Mini-Mental State Examination (MMSE) score of 21/30, a Montreal cognitive assessment (MoCA) score of 17/30, and an intelligence quotient (IQ) of 80, with failure in executive functions, processing speed, working memory, and semantic memory. During the interview, she showed emotional lability and moria. Exploration of the cranial nerves was unremarkable, and the patient presented with spastic paraparesis, generalized hyperreflexia, and cerebellar ataxia. Hormone tests showed an abnormal follicle-stimulating hormone (FSH) at 49.84 mU/mL (normal values: follicular phase = 3.5-12.5 mU/mL, ovulatory phase = 4.7-21/5 mU/mL, and luteal phase = 1.7-7.7), abnormal estradiol levels at 45.8 pg/ml (normal values: follicular phase = 173-315 pg/ml, ovulatory phase = 317-525 pg/ml, and luteal phase = 294-752 pg/ml), and normal luteinizing hormone at 20.09 (normal values: woman follicular phase = 2.4-12.6 mU/mL, ovulatory phase = 14.0-95.6 mU/mL, and luteal phase = 1.0-11.4 mU/mL). Antinuclear antibodies (ANA), perinuclear anti-neutrophil cytoplasmic antibodies (ANCA-p), anti-neutrophil cytoplasmic antibodies (ANCA-c), anti-Ro/SSA (Sjogren's syndrome-related antigen A), anti-LA/SSB (Sjögren's syndrome-related antigen B), Scl-70, anti-Jo-1, IgG-IgM anti-cardiolipin, and anti-beta-2-microglobulin antibodies were unremarkable. Paraneoplastic workup was also unremarkable. Brain MRI 1.5T was obtained, showing subcortical and periventricular white matter lesions that appear hypointense in T1-weighted images, and hyperintense on T2-weighted and fluid-attenuated inversion recovery (FLAIR). FLAIR images showed cystic degeneration within diffuse hyperintense white matter affection (Figure 1).

A leukodystrophy and leukoencephalopathy panel of 728 genes was constructed, with two pathogenic homozygous variants c338>A (p.Arg113His) in exon 3 of the EIF2B5 gene. A white matter vanishing disease was diagnosed based on classic clinical history, clinical examination findings, radiological features, and genetic confirmation. The patient was discharged with a cognitive rehabilitation workshop plan and genetic and family counseling since no definitive treatment has been established to date.

## Discussion

Leukodystrophies are a diverse category of uncommon hereditary illnesses defined by selective involvement of the brain white matter. These illnesses can affect individuals of various ages [1]. The history of these kinds of leukodystrophies is not so old. The first official description of the disease is by Eicke in 1962 where he described a 36-year-old female with progressive gait troubles and secondary amenorrhea [2]. In 1976, Deisenhammer and Jellinger described autopsy findings of severe cystic leukoencephalopathy in a young female patient with progressive spastic paraparesis and ataxia, including periods of altered consciousness and occasional seizures [3]. In 1993, Hanefeld described a triad of cases that shared similar clinical characteristics, showing progressive cognitive and gait impairments [4]. In 1997, van der Knaap associated patients with triggering stressful factors to the disease and performed MRI concluding equivocal involvement of a white matter rarefied with progressive cystic degeneration and proposed the name “vanishing white matter” [1]. The distribution of age has been described by Hamilton and colleagues in a longitudinal multicenter study with 296 vanishing white matter disease (VWMD) patients with genetic mutations confirmed, with a median age at onset of three years. Of the patients, 87% had an onset at <18 years, and 13% had an onset at >18 years old. They also described a sex prevalence ratio of 1:1.21 for males and females, with a female predominance in the adult-onset group [5]. Few infantile-onset cases have been reported in the Mesoamerican [6,7] and South American populations. Varying clinical features include spastic paraparesis, gait instability, cerebellar ataxia, tremors, weakness, psychiatric manifestations, and cognitive impairment. After disease onset, symptoms progress in some cases leading to severe disability and even death with some exceptions. In the case of concomitant ovarian failure presentations, there are milder neurological disturbances, slower progression, and slower grades of incapacity [8]. Another multicentric study with 16 patients from 14 unrelated families showed a sex ratio imbalance (male/female = 3/13), and a mean age of onset of 31.1 years (range = 16-62 years). Out of 13 females, 62% had ovarian failure, two had primary amenorrhea, five had early menopause, and one had infertility [9]. The most recent and largest overview of adult-onset VWMD cases reported in 2019 by Wei et al. presented 33 patients, of which 24 (72.7%) exhibited ovarian failure, 13 (54.2%) had secondary amenorrhea, seven (29.2%) had primary amenorrhea, three (9.1%) presented with delayed menarche, and one had infertility; ovarian failure was described preceding the neurological symptoms [10]. Our patient had menometrorrhagia, irregular menstrual cycles, and infertility; after a workshop hormonal testing, ovarian failure was confirmed, and she exhibited neurological features compatible with the classic presentation of the disease. Brain MRI [11] has its own diagnostic criteria for VWMD and shows an abnormal signal that can affect the entire white matter. Among the literature, brain MRI has described a white matter rarefaction with an increase in signal evidence on T2-weighted and FLAIR images, showing cystic degeneration usually in a bilaterally symmetric pattern. Other findings in T1-weighted, FLAIR, and proton density-pondered images showed a radiating, stripe-like pattern within the rarefied white matter, which might have been the remaining white matter strands. Contrast enhancement is not characteristic; if present, it constitutes a red flag to rule out other mimic presentations [12,13] like mitochondrial leukodystrophies. The brain MRI of our patient showed an abnormal diffuse signal of almost all white matter, with a high signal on T2-weighted, proton density, and FLAIR images, with cystic degeneration, without contrast enhancement, consistent with the WMVD radiological obligatory criteria. The five genetic mutations (EIF2B1-5) encoding the EIF2B subunits are a protein biosynthesis complex, associated with autosomal recessive leukoencephalopathy, known as VWMD [14,15]. The activity of the ElF2B guanine nucleotide exchange factor is regulated primarily by its own phosphorylation, which leads to faster protein synthesis [15]. Wei et al. found a c.338 G > A mutation in EIF2B5 (p.Arg113His) in 12 patients (36.4%), including 11 homozygous patients [10]. In our patient, a c.338 G > A mutation in the EIF2B5 gene (p.Arg113His) was confirmed by genetic testing. Although no curative treatment has been approved to date, strategies have been proposed to avoid stressors, such as moderate-to-severe infectious processes, and it has been proposed to avoid frightening, since they have been associated with the worsening of the disease [13].

## Conclusions

The adult-onset presentation of WMVD with ovarian failure constitutes a low frequency of rarely reported disease in the literature, with the largest reported cohort of only 33 cases. We consider this case valuable for the scientific community, to increase awareness about the disease, and to broaden the knowledge of the clinical phenotype, genetic background, and its behavior in Mesoamerican populations. To the best of our knowledge, this is the first report in Central America, and the first in El Salvador.
